# Effects of Krankcycle Training on Performance and Body Composition in Wheelchair Users

**DOI:** 10.1515/hukin-2015-0093

**Published:** 2015-01-12

**Authors:** Rostislav Čichoň, Adam Maszczyk, Petr Stastny, Petr Uhlíř, Miroslav Petr, Ondřej Doubrava, Aleksandra Mostowik, Artur Gołaś, Paweł Cieszczyk, Piotr Żmijewski

**Affiliations:** 1 Charles University in Prague, Faculty of Physical Education and Department of Anatomy and Biomechanics, Laboratory of Extreme Loading; 2 Department of Theory and Practice of Sport, Jerzy Kukuczka Academy of Physical Education in Katowice, Poland; 3 Palacky University in Olomouc, Faculty of Physical Culture, Tr. Miru 115, post. 771 11 Olomouc, Czech Republic; 4 Faculty of Physical Education and Health Promotion, University of Szczecin, Poland; 5 Department of Physiology, Institute of Sport, Warsaw Poland

**Keywords:** Krankcycle, wheelchair user, paraplegia, body composition, training methods

## Abstract

Innovation in training equipment is important for increasing training effectiveness, performance and changes in body composition, especially in wheelchair users with paraplegia. The main objective of a workout session is to induce an adaptation stimulus, which requires overload of involved muscles by voluntary effort, yet this overload may be highly influenced by the size of the spinal cord lesion. Krancykl construction is designed to allow exercise on any wheelchair and with adjustable height or width of crank handles, where even the grip handle may be altered. The aim of this study was to determine the differences in body composition, performance and the rate of perceived exertion (RPE) in paraplegics with a different level of paralyses after a 12 week training programme of a unilateral regime on Krankcycle equipment (a crank machine). The study sample included four men and one women at a different spine lesion level. The 12 weeks programme was successfully completed by four participants, while one subject got injured during the intervention process. Three participants were paraplegics and one was quadriplegic with innervation of the biceps humeri, triceps humeri and deltoideus. The Krankcycle 30 min programme was followed by four other exercises, which were performed after themselves rather than in a circuit training manner as the latter would result in much longer rest periods between exercises, because paraplegics have to be fixed by straps during exercise on hydraulic machines. The RPE after the workout decreased following the twelve week adaptation period.

## Introduction

Innovation in training equipment is important for increasing training effectiveness, performance and changes in body composition, especially in wheelchair users with paraplegia. Paraplegics have limited possibility for variations of exercises and also a reduced amount of muscle mass for hypertrophy stimulation.

Paraplegics are wide users of hand bikes for recreational and sport purposes, where different kinds of crank machines are used for both aerobic and anaerobic training. These two types of training are beneficial for fat percentage reduction and physical performance improvement, moreover, combination of both types of training may be implemented in one workout session (Holviala et al., 2010). Since hand bikes and traditional crank machines do not have lot of variations, combined aerobic-anaerobic training may be another way in paraplegics to increase exercise effectiveness (Smith et al., 2002). On the other hand, each training programme requires a certain period of time to induce a physiological response or body composition changes which usually lasts between 6 to 12 weeks (Havlíčková et al., 2004; Wilmore and Costil, 2004). This period is considered as the minimal, short term effect (Barfield et al., 2012; Davis and Shephard, 1990).

The key point in a workout session is to induce an adaptation stimulus which requires an overload of involved muscles by voluntary effort, yet this overload may be highly influenced by the size of the spinal cord lesion. Krankcycle construction is designed to allow exercise performed on any kind of a wheelchair with adjustable height or width of crank handles, where even grip handles may be altered. Beyond that, the handles may act as a solid or divided element with adjustable resistance, thus both bilateral or unilateral loading can be applied. The Krankcycle might seem to be a good solution for a crank machine, which provides enough individual loading variability, improves muscle coordination and performance, as well as eliminates imbalances

The aim of this study was to determine the differences in body composition, performance and the rate of perceived exertion (RPE) in paraplegics with different levels of paralyses after a 12 week training programme under a unilateral regime on Krankcycle equipment (a crank machine). We hypothesized that this type of training would increase lean body mass and enhance anaerobic as well as aerobic performance (cardiovascular variables). Additionally, there was an expectation for improvement of upper limb flexibility considering that unilateral loading variations were used in the training programme.

## Material and Methods

### Experimental Approach to the Problem

A four case study in a qualitative quasi-experimental multifactorial design was performed in a laboratory environment and each participant had a different degree of paralyses. The effectiveness of the training programme was evaluated by a standardized pre-test and post-test. The results were considered as expertize rather than strict statistical interpretation owing to a small sample size.

### Participants

The participants consisted of four men and one women of a different spine lesion level described in Table 1. The subjects were recruited in the “Paraple” centre and covered different calendar age (28–49 years), lesion ages (1–30 years) and sports experiences. Three participants were paraplegics and one was a quadriplegic with innervation of the biceps humeri, triceps humeri and deltoideus. Informed written consent was provided by each participant and the testing protocol was approved by the local Committee of Ethics in accordance with the ethical standards of the Declaration of Helsinki of 1983. One participant (Table 1) was excluded from the post-test due to injury.

### Procedures

Before the first testing session (pre-test) participants performed some exercises to become familiar with training on the Krankcycle machine. Afterwards, the pre-test took place in the laboratory followed by a 12 week training programme which included exercises on a Krankcycle machine lasting 30 min, twice per week, thus, the participants performed a total of 24 sessions. The post-test laboratory session took place one week after completing the training protocol.

The laboratory visit started with anthropometry measurements, followed by spirometry measurements and a warm up before the physical performance tests. The physical battery tests started with coordination, maximal speed and muscle endurance evaluation, followed by the 30 s Wingate anaerobic test, with minimal 10 min rest between following tests. The flexibility test was performed the next day as a separate screening tool to assess muscles stiffness. The first and last work out session were recorded for the heart rate and the RPE each 5 min of the session.

Each work out itself started with a 5 min warm up, followed by a 60 min work out protocol and stretching. The work out protocol started with 30 min continuous loading on the Krankcycle machine with resistance that allowed to keep crank frequency between 65 to 75 revolutions per minute, followed by four more exercise stations. The stations were performed on hydraulic resistance machines for dips, military press, alternated rowing and the latissimus pull down. Military press and dips were performed in 4 sets of 6 to 10 repetition maximum effort with a 2 min rest interval. Alternated rowing and the alternated latissimus pull down were performed as 5 min of continuous loading with resistance keeping the heart rate above the individual anaerobic threshold.

### Measures

#### Anthropometrics

Body height was measured in the prone position by a digital stadiometer (SECA 242, Hamburg, Germany), body mass was measured by a digital scale (SECA 769, Hamburg, Germany) to calculate the body mass index (BMI). The circumferences of the forearm, arm, waist and hips were measured by an anthropometric tape measure and the waist to hip ratio (WHR) index was calculated. Shoulder flexibility was assessed by the distance (cm) between fingers in a shoulder joint reach flexibility test. Elbow flexibility was assessed by both procesus styloideus ulnae distance in elbow extension with both medial epicondyle humeri in contact.

Body composition was assessed by bioelectrical impedance (Bodystat QuadScan 4000, Bodystat, Douglas, United Kingdom).

#### Spirometry

Respiratory functions such as forced vital capacity (FVC), forced expiratory volume in 1 second (FEV1) and the peak expiratory flow (PEF) were estimated by Pony Graphic (Cosmed srl., Italy) and expressed in absolute values and percent of proper values (ECCS 1983).

#### Physical performance

Anaerobic performance was estimated by the 30 s Wingate test for upper limbs (Hutzler, 1998). The test was performed on a mechanical crank with XY with an individual load in the range of 1 – 2 Watts per BM (W·kg^−1^) fitted in relation to age, training experience and the level of the spinal cord lesion to achieve a work zone between 50 – 55 revolutions per minute (Heller and Příbaňová, 2000). Peak power (PP) was estimated from the best 5 s of the test and expressed in absolute and relative values. Mean power (MP) was estimated in absolute values and as anaerobic capacity (AnC), where AnC was counted as the product of MP and test time. The fatigue index (FI) was counted as the PP/minimal power ratio expressed in percentages. The index of speed/endurance was calculated as the MP/PP ratio expressed in percentages. The heart rate was recorded by the telemetric method (Sport-Tester PE 4000, Polar Electro, Finsko). The post-test blood lactate concentration was evaluated by the electrochemical device Biovendor Super GL from a fingertip capillary sample 5 min after test cessation (Smith et al., 2002).

Strength endurance was evaluated by a modified test used previously by Barfield et al. (2012) which consisted of the maximal number of repeated full elbow flexion/extension with a 2.5 kg dumbbell in 30 s. This test was performed unilaterally on both upper limbs. The coordination test was performed according to Rodgers et al. (2001) on the Krankcycle machine, where the fluency of pre-defined movements was estimated in two attempts with a 3 min rest interval. The best attempt was evaluated qualitatively. The speed test was conducted on the Krankcycle machine with an adjusted marginal load with the instructions to achieve the highest revolutions per minute.

#### Response to the training protocol

The Borg rate of perceived exertion (RPE) 6 to 20 scale was used to assess the voluntary effort during the training protocol, which was proportional to the Krankcycle work out (Boyer, 2009). This analogue scale measures relative perception of effort in ranges from 6 (very, very light) to 20 (very, very hard). Every 5 min the participants were asked to indicate a number on a printed chart displaying the RPE scale. The values were then manually recorded. The heart rate was recorded by telemetry (Sporttester PE 3000, Polar Electra, Kempele, Finland) during each testing and workout protocol, to estimate the overall physiological demand. The heart rate during Krankcycle training was previously reported even higher than during testing sessions (Boyer, 2009).

### Results interpretation

Analysis of the obtained data was performed by two experienced paraplegics practitioners. The expertise approach in evaluating was used considering previous findings that maximal values might be substantially lower than expected with considerable variability among paraplegics in the metabolic responses to maximal exercise (Cooper et al., 1992). Improvements in performance of 10% or greater were considered significant, however, expertise constituted the main criteria.

## Results

Body composition changes were found only in participant 4 (Table 2), who increased his LBM and decreased the percentage of body fat by 4.4%. This change was accompanied by an increase in cellular mass and fat free mass. An increase in arm circumference was found in the right upper limb of participants 1, 2 and 3, and the left upper limb of participant 4 (Table 2). Forearm circumference increased in the right upper limb of participants 2, 3 and 4, and in the left upper limb of participant 3 (Table 2).

Respiratory measurements showed an increase in all recorded variables in participant 4, but a decrease in all respiratory variables in participant 3 (Table 3). Participant 1 increased both the absolute and relative PEF (Table 3). Participant 2 increased both absolute and relative FEV1 and PEF (Table 3).

## Discussion

The 12 week training program was successfully performed by four participants, while one got injured during the intervention process. However, the paraplegics had difficulty in maintaining their health for prolonged time, therefore, completion of all 24 work out sessions by four participants was considered as success. Since there were two workout sessions per week, work out frequency may be increased to reach better results in future experiments.

The Krankcycle 30 min programme was followed by four other resistance exercises, which were performed after themselves rather than in a circuit manner as it would result in much longer rest periods between the exercises, taking into account that the paraplegics had to be fixed by straps during work on the hydraulic machines. The RPE of the workout decreased due to the 12 week adaptation to the exercise programme of which duration was in line with the results of previous studies (Siff, 2003; Hicks et al., 2011).

Participants 1 and 2 improved their respiratory functions such as PEF (in %) to the level which had been previously reported in the study performed on participants with a similar level of the lesion (Yim et al., 1993). This previous study did not find improvements in respiratory functions after the training programme, however, their post-training level was above 90% of predicted values (Yim et al., 1993). The respiratory function varies due to the level of the spinal cord lesion (Baydur et al., 2001; Van Loan et al., 1987), yet participants 1, 2 and 3 presented above average values of FEV in the post-test compared to baseline values (Baydur et al., 2001). On the other hand, FEV1 and FVC were found to be independent of the training level (Zwiren and Bar-Or, 1974), which was not confirmed in participant 2.

Physical performance assed by the 30 s Wingate test increased in participant 2 and decreased in participant 4. Participant 4 had the lowest anaerobic performance at the pre-test and it was suggested that the subject was overtrained. It was not possible to compare the WG test results with previous studies on paraplegics, as previous data reported only absolute values (Nash et al., 2007) while in our study only relative values were presented. However, both WG 30 tests were performed in accordance with recommendations for paraplegics (Jacobs et al., 2002).

Flexibility increased in all participants, which may be the cause of unilateral loading used for the Krankcycle workout. An increase of flexibility had been reported after the strengthening and flexibility programme (Rodgers et al., 2001) with the use of 3D measurements during functional movement rather than individual flexibility tests.

All participants had more LBM than it is generally referred for paraplegics (Spungen et al., 2003; Maggioni et al., 2003), but these values are not referred for athletes or an active population. The arm and forearm circumferences were similar to the paraplegic athletes population (Bulbulian et al., 1987) besides participant 4 who should be considered more likely as a quadriplegic. Body composition changes after training in paraplegics were found very difficult to achieve along with enhancement in physical capacity and muscular strength (Hicks et al., 2011).

The main limitation of this study includes a low number of participants. The reason is that only a small number of paraplegics decided to volunteer for the full 12 week training programme, which is understandable considering real difficulty in maintaining appropriate health status in this population. Another difficulty is lack of normative values for most tests for this population.

## Conclusion

The Krankcycle training was found effective in inducing body composition change and enhancement of physical performance in wheelchair users, where each participant improved different variables following individual training conditions. A significant adaptation to the Krankcycle training program was recorded after 12 weeks, thus, the authors suggest further modifications of the exercise load, frequency and order after this time period.

## Figures and Tables

**Table 1:** Basic data of the participants

Participant	Gender	Age (years)	Body heigh (cm)	BM (kg)	Lesion level	Lesion age (years)	Dominant limb	Paralyze reason
1	male	39.6	192	108.2	Th_12_-L_4_	14.7	right	High fall
2	female	48.7	179	57.2	Th_10_	1.1	left	High fall
3	male	27.7	173	83	Th_8_	15.4	right	High fall
4	male	21.2	190	59	C_5_	1.7	right	Car accident
5	male	47.3	180	116.7	Th_5_	30.2	right	Sport injury
average		36.9 ± 11	182.8 ± 7	84.8 ± 25		12.6 ± 11		

**Table 2:** Anthropometric variables and body composition changes

Variable	Participant 1			Participant 2			Participant 3			Participant 4		
	pre	post	Δ	pre	post	Δ	pre	post	Δ	pre	post	Δ
Body mass	108.2			57.2			83			59		
Body Fat (%)	26.3	27.7	1.4	18.2	19.6	1.4	18.2	20.0	1.8	11.7	7.3	4.4^*^
LBM (kg)	79.9	77.1	2.8	46.6	45.8	0.8	67.9	66.4	1.5	52.1	58.8	6.7^*^
Anhydrous (kg)	25.6	25.8	0.2	12.2	11.9	0.3	20.9	21.1	0.2	14.3	16.6	2.3^*^
Total water (%)	50.0	48.2	1.8	60.4	59.5	0.9	56.6	55.3	1.3	64.1	66.6	2.5
ETC (%)	21.8	21.2	0.6	28.3	28	0.3	24.1	23.3	0.8	27.9	28.8	0.9
ITC (%)	27.4	27	0.4	29	29	0	30.7	31	0.3	33.5	34	0.5
Cellular mass (%)	42.4	40.8	1.6	23.6	23.2	0.4	36.4	36.2	0.2	28.2	30.9	2.7^*^
3 space (l)	0.8	0.4	0.4 ^*^	1.8	1.7	0.1	0.9	0.6	0.3	1.5	2.3	0.8
BMI	29.4	29.1	0.3	17.9	17.9	0	27.7	27.7	0	16.3	17.6	1.3^*^
WHR	0.92	0.92	0	0.87	0.86	00	0.89	0.90	0	0.83	0.83	0
R Arm circ. (cm)	37	39	2^*^	28	30	2^*^	37	39	2^*^	24	25	1
L Arm circ. (cm)	38	39	1	29	30	1	38	39	1	23	25	2^*^
R Forearm circ. (cm)	34	34	0	26	27	1^*^	32	33	1^*^	21	22	1^*^
L Forearm circ. (cm)	34	34	0	27	27	0	32	33	1^*^	22	22	0
Waist circ. (cm)	120	122	2	78	78	0	93	94	1	72	71	1
Hip circ. (cm)	130	130	0	90	91	1	105	104	1	86	86	1

**Table 3:** Pre-test and post-test respiratory variables

Variable	Participant 1			Participant 2			Participant 3			Participant 4		
	pre	post	Δ	pre	post	Δ	pre	post	Δ	pre	post	Δ
FVC (% p.v.)	85	84	1	72	74	2	76	67	9^*^	42	49	7^*^
FVC (l)	4.83	4.76	0.07	2.71	2.78	0.07	3.72	3.30	0.42^*^	2.52	2.90	0.38^*^
FEV1 (% n.h.)	87	83	4	75	84	9^*^	88	78	10^*^	45	50	5^*^
FEV1 (l)	4.03	3.85	0.18	2.46	2.73	0.27^*^	3.66	3.26	0.40^*^	2.24	2.48	0.24
PEF (% n.h.)	74	108	34^*^	77	106	29^*^	81	71	10^*^	31	37	6^*^
PEF (l.s-1)	7.59	11.13	3.54^*^	5.60	7.70	2.10^*^	7.76	6.78	0.98^*^	3.31	3.99	0.68^*^

FVC = forced vital capacity, FEV_1_ = forced expiratory volume in 1 second, PEF = peak expiratory flow, Δ = delta, difference between pre-test and post-test in absolute values, pre = pre-test, post = post-test.

**Table 4:** Differences in physical performance

Variable	Participant 1			Participant 2			Participant 3			Participant 4		
	pre	post	Δ	pre	post	Δ	pre	post	Δ	pre	post	Δ
PP (W·kg^−1^)	4.1	4.2	0.1	3.8	4.1	0.3^*^	4.6	4.6	0	1.6	1.5	0.1
MP (W·kg^−1^)	3.2	3.2	0	3.1	3.4	0.3^*^	3.6	3.7	0.1	1.2	1.1	0.1
AnC (J·kg^−1^)	96	94.6	1.4	92.2	102.6	10.4^*^	109.1	110.3	1.2	36.3	31.6	4.7
FI (%)	50.3	55.4	5.1^*^	31.8	28.9	2.9^*^	42	39.9	2.1	40.5	50.5	10^*^
MP/PP (%)	78.1	74.3	3.8	80.9	84.1	3.2	78,6	80.5	1.9	77.4	70.6	6.8^*^
HRmax	179	166	13^*^	155	161	6	157	146	11^*^	118	144	26^*^
LA (mmol·l^−1^)	10.6	11.4	0.8^*^	6.8	10.3	3.5^*^	7.9	8.5	0.6	5.8	5.6	0.2
Endurance RA	24	27	3^*^	24	28	4^*^	24	28	4^*^	15	16	1
Endurance LA	23	27	4^*^	26	29	3^*^	22	26	4^*^	12	15	3^*^
coordination	26.9	26.3	0.6	25.8	25.8	0.8	29.2	28.4	0.8	33.6	31.1	2.5
speed	94	98	4	81	86	5^*^	68	75	7^*^	40	49	9^*^

Δ = delta, difference between pre-test and post-test in absolute values, pre = pre-test, post = post-test, PP = peak power, MP = mean power, AnC = anaerobic capacity, FI = fatigue index, MP/PP = , HRmax = heart rate, LA = lactic acid.

**Table 5:** Differences in the rate of perceived exertion on the Borg scale (6–20)

Variable	Participant 1			Participant 2			Participant 3			Participant 4		
	pre	post	Δ	pre	post	Δ	pre	post	Δ	pre	post	Δ
Right shoulder (cm)	13	10	3^*^	0	−1	1^*^	17	15	2^*^	6	2	4^*^
Left shoulder (cm)	1	0	1^*^	7	3	4^*^	20	17	3^*^	1	0	1^*^
Elbow	40	42	2^*^	46	49	3^*^	18	22	4^*^	47	48	1

Δ = delta, difference between pre-test and post-test in absolute values, pre = pre-test, post = post-test,												

time (min)	Participant 1			Participant 2			Participant 3			Participant 4		
	pre	post	Δ	pre	post	Δ	pre	post	Δ	pre	post	Δ

0	16	12	4^*^	15	13	2^*^	10	13	3^*^	8	8	0
5	18	12	6^*^	13	14	1	12	14	2	17	10	7^*^
10	19	15	4^*^	14	14	0	12	14	2	12	8	4^*^
15	19	16	3^*^	16	13	3^*^	13	13	0	10	12	2
20	17	11	6^*^	15	12	3^*^	14	12	2	10	12	2
25	16	10	6^*^	15	12	3^*^	12	12	0	12	14	2
30	14	10	4^*^	14	12	2	10	10	0	12	12	0

Pre = pre-test, post = post-test.

Heart rate did not differ between the initial and final work out session.
